# A profile of psychotropic medication in bipolar patients of reproductive age

**DOI:** 10.4102/sajpsychiatry.v32i0.2625

**Published:** 2026-05-19

**Authors:** Nkateko P. Matoba, Sibusiso N. Sotobe Mose, Tiaan Schutte

**Affiliations:** 1 Department of Psychiatry, Faculty of Health Sciences, University of the Witwatersrand, Johannesburg, South Africa

**Keywords:** mental health, mental illness, medication, bipolar disorder, females, reproductive age, treatment, treatment guidelines

## Abstract

**Background:**

Bipolar disorder (BD) is associated with unfavourable reproductive health outcomes. Evidence-based treatment guidelines advocate for reproductive safe prescribing, including family planning, avoidance of teratogenic medication and documentation of risk acknowledgement. However, there is poor adherence to the prescribed guidelines in clinical practice.

**Aim:**

To describe the range of psychotropic medications prescribed to women of reproductive age (WRA) with BD at a tertiary psychiatric unit.

**Setting:**

Helen Joseph Hospital, Johannesburg, South Africa.

**Methods:**

A retrospective record review of females aged 18–49 years treated for BD between January 2016 and December 2023 was conducted. A convenience sample of 141 files was reviewed. Data were collected from the latest clinical notes and prescription charts.

**Results:**

A combination of mood stabilisers (MS) and atypical antipsychotics was predominantly prescribed (81.6%). Of the MS, valproate use was most common (50.6%), followed by lithium (29.3%). Risperidone was the most prescribed antipsychotic (39.0%), followed by olanzapine (24.7%) and quetiapine (20.5%). Antidepressants were combined with MS or antipsychotics in 10.6% of cases. Documented contraception counselling (5%) and contraception use (9.2%) were low. None of the records had documented risk acknowledgement for valproate use (100%).

**Conclusion:**

Prescribing guidelines for teratogenic MS were not consistently followed. Therefore, WRA diagnosed with BD may be at an increased risk of teratogenic drug exposure in pregnancy.

**Contribution:**

This study highlights the need for improved documentation of medication risk discussions, family planning and informed consent at every visit.

## Introduction

Bipolar disorder (BD) is a diverse mood condition with a lifetime prevalence of 1% – 2%.^1,2^ It is characterised by episodic manic, hypomanic and depressive mood states.^3^ Despite similarity in the prevalence across all genders, treatment in women requires special considerations.^1,2^

The onset of BD is commonly in the late teens and early 20s, a period coinciding with pregnancy for many women.^2^ Approximately 35% of women diagnosed with BD are of reproductive potential.^4^ The World Health Organisation (WHO) defines women of reproductive age as those aged 15–49 years.^5,6^ Nearly half of all pregnancies are unplanned, thus risking exposure to potentially teratogenic medication.^4^ Moreover, BD is associated with unfavourable pregnancy outcomes such as hypertensive conditions, antepartum haemorrhage, pre-term delivery, small birth weight, assisted and caesarean section deliveries.^7,8^ The risk of mood episode relapse in the peripartum period is significant and is greater in women not on treatment.^7,8^ Therefore, counselling on family planning and risks associated with some psychotropic agents is essential for all women of reproductive age (WRA) at every visit.^1,8,9,10^ Consequently, reproductive-safe prescribing has drawn increasing attention as a means to optimise treatment outcome in women.^1,9,10^

Medication is the mainstay of treatment for BD and is required to prevent relapse.^1,9,10,11^ Mood stabilisers (MS): lithium and anticonvulsants (valproate, lamotrigine and carbamazepine) and antipsychotics are indicated.^1,8,9,10,11^ Antidepressants, though controversial, play a role in bipolar depression.^1,8,10,11^ In WRA, the risk–benefit assessment is crucial, with consideration given to the most effective and safest medication.^1,9,10,12^

### Treatment of acute manic episodes

Generally, treatment is individualised according to various factors such as history of the illness course, treatment response and tolerability, adherence profile, comorbidities and availability.^1,9,10,11^ Medications effective across different bipolar phases are preferred.^1,9,10,11^ The South African National Department of Health Standard Treatment Guidelines (STG)^9^ recommend lithium monotherapy as first-line treatment in acute mania.^9^ The second-line options are valproate, olanzapine and risperidone.^9^ In contrast, the National Institute for Health and Care Excellence (NICE)^1^ guidelines recommend antipsychotics as first-line treatment.^1^ National Institute for Health and Care Excellence endorses olanzapine, quetiapine and risperidone but regards lithium and valproate as second-line options.^1^

The greatest consensus exists between the South African Society of Psychiatrists (SASOP),^11^ the Canadian Network for Mood and Anxiety Treatments (CANMAT) and International Society for Bipolar Disorders (ISBD),^10^ both MS (lithium and valproate) and antipsychotics are recommended as first-line interventions.^10,11^ The following atypical antipsychotics are effective in a manic episode: risperidone,^1,10,11^ quetiapine,^1,10,11^ aripiprazole,^10,11^ paliperidone,^10,11^ asenapine^10^ cariprazine^10^ and ziprasidone.^11^ However, the use of the latter three agents is scarce in South Africa because of no availability in the public sector.^9^ Although haloperidol is effective in acute mania, the risk for extrapyramidal side effects and poor efficacy in relapse prevention limits its use.^1,9,11^ Consensus remains elusive on the use of carbamazepine in manic disorders. It is recommended by the CANMAT guidelines as a second-line agent while the local STG reports no efficacy in any mood episode.^9,10^

Monotherapy treatment with first-line agents is effective in roughly half of the patients.^10^ However, combination therapy of lithium or valproate and an atypical antipsychotic is generally preferred because of the evidence of higher efficacy and a more rapid response, despite its cumulative side effect risk.^8,10^ This is an important factor in resource-limited settings where rapid recovery is desirable.^13^

### Treatment of acute bipolar depression

Bipolar I disorder (BD I) has at least one manic episode lasting ≥ 7 days (or requiring hospitalisation), often with psychosis and major impairment, with or without depressive episodes. Bipolar II disorder (BD II) is defined by at least one hypomanic episode—lasting ≥ 4 days and characterised by an elevated or irritable mood without marked functional impairment or psychosis—and at least one major depressive episode. BD I and BD II are distinct conditions with unique treatment requirements.^9,10,11^ The STG recommends lithium, valproate and olanzapine as first line for acute BD I depression depending on tolerance and response to them.^9^ Additionally, quetiapine, lamotrigine and lurasidone monotherapy are endorsed by CANMAT and ISBD and SASOP.^10,11^ Lurasidone and lamotrigine are effective as add-on therapy in cases of lithium-poor response.^10^ Quetiapine and lamotrigine may be added to lithium or valproate in cases of relapse while on optimal doses.^9,10,11^

There is no consensus regarding the treatment of acute BD II depression.^9,10^ The Canadian guidelines recommend quetiapine as first-line treatment because it is effective in both BD I and BD II depression.^10^ In contrast, lithium therapy is the first-line treatment in the local STG.^9^ Lamotrigine and quetiapine are the alternatives in cases of lithium non-response.^9^ Lamotrigine requires slow titration because of the risk of a severe drug reaction, thereby causing delays in achieving clinical response.^9,10,11^

The role of antidepressants in BD depression is not yet confirmed.^1,8,9,10,11^ Antidepressant monotherapy is not recommended in BD I depression because of the risk of a manic switch.^1,8,11^ National Institute for Health and Care Excellence guidelines recommend a combination of olanzapine and fluoxetine as first-line treatment therapy because of its greater efficacy over lamotrigine monotherapy.^1^ This combination is recommended as a second line by others^10,11^ and demonstrates no efficacy in BD I depression prophylaxis.^9,11^

The CANMAT and ISBD guidelines recommend bupropion, sertraline and venlafaxine monotherapy as second-line options for acute BD II depression, while venlafaxine and fluoxetine are recommended for prophylaxis.^10^ Notably, these recommendations are based on studies in pure depression,^10^ whereas bipolar depression typically presents with atypical or mixed features,^3^ in which the role of antidepressant monotherapy is ambiguous. In contrast, NICE, STG and SASOP advise against using antidepressant monotherapy for BD I or II depression because of associated uncertain benefits and mood switch risk; if used, these antidepressants should be combined with a MS, and mood is monitored closely.^1,9,11^

### Bipolar disorder maintenance treatment

Generally, agents effective in the acute phase are continued in the maintenance phase.^1,9,10^

Lithium presents the best evidence for mania and depression prophylaxis, with a unique anti-suicidal effect.^1,8,9,10^ Other agents, such as valproate, quetiapine, asenapine and aripiprazole (oral or injectable), are recommended for the prevention of mania and lamotrigine for the prophylaxis of depression.^1,10^ The combination of quetiapine and lithium or valproate has also proven effective.^1,10,11^

Lastly, depot antipsychotics play a role in cases of poor adherence.^1,8,11^ Risperidone long-acting injectable (LAI) bi-weekly monotherapy or as an adjunct and aripiprazole LAI are effective against mania but not depression.^10,11^

### Treatment implications in women of reproductive age

Monotherapy prophylaxis is recommended at the lowest effective dose when feasible.^10^ Treatment should be individualised while prioritising safety and efficacy.^1,10,11^ Antipsychotics are considered to be the safest option in pregnancy and breastfeeding, especially quetiapine.^9^ The local STG recommends olanzapine because of efficacy in both mania and depression.^9^

The risk of weight gain and diabetes in pregnancy is maximum with olanzapine intake.^9^ Therefore, the Canadian guidelines advised olanzapine use for second-line treatment.^10^ One study reported an increased congenital risk associated with risperidone, thus not for first-line use in pregnancy.^14^ Additionally, risperidone may increase prolactin levels in non-pregnant WRA, which may impair fertility.^10^

Regarding MS, carbamazepine is associated with a risk of neural tube abnormalities and therefore not recommended in pregnancy.^1^ Lithium and valproate are teratogens associated with congenital malformations.^15,16^ Lithium is associated with a 2.4 cases per 100 live births risk of cardiac defects if intra-uterine exposure occurs in the first 3 months; however, this risk is lower than previously suggested.^9,15^ Furthermore, lithium requires regular blood level monitoring, more so during pregnancy because of its narrow therapeutic index.^8,9^ It is excreted in breastmilk and has a risk of toxicity in the infant, thereby making it unsuitable during breastfeeding.^8,9^

Valproate is contraindicated in WRA because of its high risk for congenital malformations and poor neurodevelopmental outcomes in exposed infants.^1,9,10,16,17^ If valproate use is unavoidable, a risk acknowledgement declaration should be signed and pregnancy prevention methods implemented.^1,8,9,10,18^ Both the South African Health Products Regulatory Authority (SAHPRA)^17^ and Sanofi^18^ recommend regular counselling on teratogenic risk and contraceptive use at every visit.^17,18^ Additionally, folic acid supplements of 5 mg/day are recommended.^19,20^

The literature demonstrates a considerable risk associated with BD in WRA.^7,8^ Hence, there is a need to follow evidence-based clinical guidelines to improve the treatment outcome in this population.^1,7,9,10^ International and local studies have highlighted a discrepancy between treatment guidelines and clinical practice in managing women with BD.^4,21,22^ Therefore, this study aims to describe the range of psychotropic medications prescribed in WRA with BD at a tertiary psychiatric unit. The documentation of discussions on effective pregnancy prevention, current contraceptive practices, prescription of folic acid and informed consent for valproate use was evaluated to determine adherence to prescribed guidelines.

## Research methods and design

### Objectives

The research objectives of this study were:

- To describe the demographics and clinical data of the study population.
- To describe the psychotropic medications prescribed in WRA with BD.
- To evaluate whether the prescribing practices align with treatment guidelines in WRA with BD.

### Study design and setting

A quantitative, retrospective record review was conducted at Helen Joseph Hospital (HJH) psychiatric unit in Johannesburg, South Africa, from 01 January 2016 to 31 December 2023. Helen Joseph Hospital (HJH) is a public general hospital under the Gauteng Department of Health, affiliated with the University of the Witwatersrand. As a tertiary institution, HJH provides a variety of specialised services, including psychiatric care, to a diverse population from different parts of the greater Johannesburg metropolitan region.

### Study population and sampling strategy

Patients’ files were screened for WRA, defined as those aged 18–49 years^5,6^ with a diagnosis of bipolar and related disorder as per Diagnostic and Statistical Manual of Mental Disorders, Fifth Edition, Text Revision (DSM-5-TR) criteria,^3^ that is, BD I (mania, depressive or mixed episode), BD II (hypomanic or major depressive episodes), substance/medication-induced bipolar and related disorder^3^ and bipolar and related disorder due to another medical condition.^3^

Females treated at the HJH psychiatric unit between January 2016 and December 2023 only were included in the study sample. Those individuals with unclear or provisional diagnosis and having records with a missing prescription chart were excluded from this study. We conveniently sampled the individuals to include in the study. We considered everyone who presented from 01 January 2016 to 31 December 2023 at the institution. Overall, 339 files were screened and only 141 met the inclusion criteria, and this is the study sample size.

### Data collection

Data were extracted from the patients’ files manually by the primary investigator, using the data capture sheet (Online Appendix 1). The following variables were extracted from the file and the latest clinical notes:

- Demographic variables including age (years), relationship status, level of education, residence and source of income.
- Clinical variables: Comorbidities and gynaecological history.
- Medication and documentation of contraception counselling.
- Documentation of risk acknowledgement for valproate use.

A 10% quality check was done to the files retrieved to check for errors and consistency in data capturing.

### Data analysis

The data for this study were captured in Microsoft Excel™ and statistically analysed in R software (version 4.00; www.R-project.org). The Shapiro–Wilk test was used to assess the normality of continuous variables. Continuous data are presented as the mean and standard deviation (s.d.), and categorical data are presented as counts and percentages. Bar charts were also used to display the distribution of the psychotropic agents.

### Ethical considerations

Ethical clearance was obtained from the Human Research Ethics Committee of the University of the Witwatersrand (Ref. no. M240347 M240430-A-0001). The requisite permission was granted by the HJH research committee. Patient confidentiality and anonymity were maintained by not recording identifying data.

## Results

### Demographics and clinical data

The mean (s.d.) age was 33.58 (s.d. = 7.69) years. Most women were single (*n* = 101, 71.6%) and were staying with their families (*n* = 116, 82.3%). The majority had a high school education (*n* = 85, 60.3%), only 31.9% (*n* = 45) were employed and 9.2% (*n* = 13) received a disability grant (Table 1).

**TABLE 1:** Socio-demographic characteristics of the study population (*N* = 141).

Variable	*n*	%
**Relationship status**		
Single	101	71.6
Married	20	14.2
Stable relationship	8	5.7
Divorced	11	7.8
Widowed	1	0.7
**Highest level of education**		
Primary school	1	0.7
High school	85	60.3
Tertiary	26	18.4
Undocumented	29	20.6
**Employment status**		
Employed	45	31.9
Unemployed	92	65.2
Undocumented	4	2.8
**Social disability grant**		
Receiving	13	9.2
Not receiving	42	29.8
Undocumented	86	61.0
**Residence**		
Stays with family	116	82.3
Stays alone	16	11.3
Care facility	1	0.7
Homeless	6	4.3
Undocumented	2	1.4

Analysis of clinical characteristics revealed that over a third of women (*n* = 59, 34.7%) had a substance use disorder, 20 (11.8%) had human immunodeficiency virus (HIV), while 10 (5.9%) had epilepsy and 6 (3.5%) had anxiety disorders (Table 2). Only one woman was pregnant, and 13 (9.2%) women were on contraception using injectables (6, 4.3%), intra-uterine device (*n* = 3, 2.1%) and oral contraceptive pill (*n* = 4, 2.8%) (Table 2). The majority of the women showed an undocumented pregnancy status (55%) and contraceptive use (79%).

**TABLE 2:** Clinical characteristics of the study population (*N* = 141).

Variable	*n*	%
**Bipolar subtype**		
BD I	111	78.7
BD II	13	9.2
SMIBRD	10	7.1
BRD-AMC	5	3.5
Undocumented subtype	2	1.4
**Comorbidities**		
Anxiety disorder	6	3.5
Epilepsy	10	5.9
HIV	20	11.8
Substance use disorder	59	34.7
Other (non-psychiatric and psychiatric)	21	12.4
None	48	28.2
Undocumented	6	3.5
**Pregnancy status at last visit**		
Positive	1	0.7
Negative	62	44.0
Undocumented	78	55.3
**Contraceptive use**		
Yes	13	9.2
No	17	12.1
Undocumented	111	78.7

BD I, bipolar I disorder; BD II, bipolar II disorder; SMIBRD, substance/medication-induced bipolar and related disorder; BRD-AMC, bipolar and related disorder due to another medical condition; HIV, human immunodeficiency virus.

### Psychotropic medication profile

**FIGURE 1:**
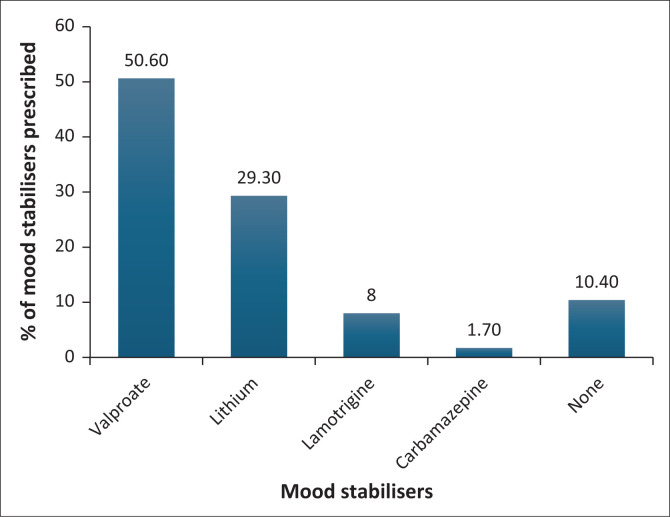
Mood stabilisers prescribed in 141 bipolar women at Helen Joseph Hospital.

Most women (*n* = 123, 87.2%) had received MS. Valproate was predominantly prescribed (*n* = 88, 50.6%) and only nine (6.4%) women received folic acid co-prescription. This was followed by lithium (*n* = 51, 29.3%), lamotrigine (*n* = 14, 8.0%) and carbamazepine (*n* = 3, 1.7%) (Figure 1). Furthermore, 133 (94.3%) women received oral antipsychotics. Some received more than one antipsychotic; therefore, the total number of antipsychotics exceeds the number of participants. Risperidone was most commonly prescribed (*n* = 57, 39.0%), followed by olanzapine (*n* = 36, 24.7%), quetiapine (*n* = 30, 20.5%), amisulpride (*n* = 5, 3.4%), haloperidol (*n* = 4, 2.7%) and aripiprazole (*n* = 3, 2.1%). Besides, 14 (9.9%) women received depot antipsychotics, predominantly flupentixol decanoate (*n* = 11, 7.8%). Only two (1.4%) women were given paliperidone and one (0.7%) zuclopenthixol decanoate. Fifteen (10.6%) women received antidepressants. Six women (4.3%) received citalopram, five (3.5%) fluoxetine, three (2.1%) duloxetine and one venlafaxine (0.7%).

Only 21 (14.9%) women received monotherapy, predominantly with atypical antipsychotics (*n* = 12, 8.5%). This includes the one pregnant woman who received olanzapine monotherapy. Mood stabilisers monotherapy was employed in five women (3.5%) and typical antipsychotics in three (2.1%).

The majority (*n* = 120, 85.1%) of women received two or more agents, predominantly a MS and an antipsychotic (*n* = 115, 81.6%). The most common combination was valproate and an antipsychotic (*n* = 58, 41.1%), followed by valproate, lithium and an antipsychotic (*n* = 26, 18.4%) and lastly lithium and an antipsychotic (*n* = 20, 14.2%). Antidepressants were prescribed with antipsychotics and/or MS (*n* = 15, 10.6%).

### Documented consent for valproate and contraception counselling

The results demonstrate that none of the women receiving valproate had documented risk acknowledgement, that is, consent (*n* = 88, 100%). Only seven (5%) women had documented counselling on contraception use at the latest consultation. These seven women, who were documented to have received counselling, later received valproate. There was no statistically significant difference in age between participants who had documented contraception counselling (median = 34.0 years, interquartile range [IQR]: 14.0) and those who did not (median = 25.0 years, IQR: 16.0), as indicated by a Mann-Whitney *U* test (*U* = 64.0, *p* = 0.164). Given the highly skewed distribution of the documented counselling, (7 vs 133 not documented), bivariate analysis across socio-demographic variables did not yield any statistically meaningful inferences. The profile of those seven women who had documented counselling is provided as a supplement (Online Appendix 2).

## Discussion

This study aims to describe the demographics, clinical data and psychotropic medication prescribed in WRA with BDs. We also evaluated documentation of discussions on effective pregnancy prevention, current contraceptive practices, prescription of folic acid and informed consent for valproate use to ascertain adherence to existing guidelines regarding valproate usage.

This study found an unemployment rate of 65% among WRA with BD. This is almost double the estimated 35.8% incidence of local female unemployment rate in the second quarter of 2024 in South Africa.^23^ Some studies observed a significant association of BD with high unemployment rates.^24^ Comorbidities are common in BD,^25,26^ and substance use disorder and HIV were predominant. Generally, comorbidities affect the illness course and at times complicate management.^25,27^ For instance, the literature notes that valproate is often preferred over lithium in patients with alcohol use disorder.^1,26^ The influence of alcohol use disorder on prescribing patterns in WRA was not specifically examined in this study.

Mood stabilisers were found to be the most prescribed psychotropic medication (87.2%). These prescription rates are similar to those of other local (83.8%)^28^ and international (72% – 90%)^29^ studies. Valproate was the most prescribed MS (50.6%), followed by lithium (29.3%). Lamotrigine and carbamazepine prescription prevalence was 8.0% and 1.7%, respectively. Lamotrigine is indicated for bipolar depression and is recommended more often in BD II than in manic predominant BD I^9,10,11^ and is the safest antiepileptic MS in terms of teratogenicity.^8^

Other studies found a much higher (31.8%) prevalence of lamotrigine prescription in BD.^28^

The lower prescribing rate in this study may reflect the lower proportion of women with BD II (9.2%). It may also indicate a clinician’s preference for agents with a faster clinical response.^9,10,11^

Atypical antipsychotics were predominantly prescribed in this study (86.3%) versus typical antipsychotics, a finding consistent with the literature.^28,30^ Atypical antipsychotics have mood-stabilising properties.^12^ So, they are endorsed by multiple guidelines as first-line agents either as monotherapy or in combination with MS.^1,9,10,11^ In the present study, risperidone, followed by olanzapine was the most prescribed agent. One international study revealed that quetiapine and olanzapine were the most popular agents.^30^ The difference may be attributed to the availability of risperidone at all levels of healthcare in South Africa, allowing patients continued access after tertiary care.^9^

Overall, 10.6% women received antidepressants in combination with MS and/or antipsychotics. This contrasts the higher prevalence reported in a previous local study in which 48.7% of participants received antidepressants.^28^ The difference may reflect the clinician’s preference for alternative agents because of the high risk for manic switch associated with antidepressants in general.

The majority of women participants received more than one psychotropic agent (*n* = 120, 85.1%). Polypharmacy is a widespread practice in BD,^31^ further supporting our study results. Over 80% of the cohort received multiple psychotropic agents in another study.^30^ A local study reported a 93.8% rate of polypharmacy in BD at a tertiary hospital.^28^ The combination of a MS and an antipsychotic was a commonly observed prescribing pattern (81.6%), consistent with findings from other studies.^28,29,30^ Both national and international guidelines support this combination, citing evidence for enhanced efficacy.^1,9,10^ Although some guidelines support combination treatment,^9,10,11^ its use in WRA requires caution to limit cumulative teratogenic risk.

Local and international guidelines^10,11^ have approved lithium and valproate as first-line agents; however, valproate should be avoided in WRA, and lithium needs careful monitoring during pregnancy.^1,9,10,17^ In cases where valproate use is unavoidable, women should be counselled (and documented) on the associated teratogenic risk and use of effective contraception at every visit.^1,9,10,17^ This was not the case in our study as not everyone prescribed valproate had documented counselling on contraception use. Only seven women on valproate had documented counselling, which confirms it was done, while the rest of the women lacked any documentation and therefore can be considered as no counselling done. Moreover, valproate interferes with folate metabolism, a vitamin essential for normal neural development.^19,20^ In such instances, folic acid at a daily dose of 5 mg is advised in people treated with valproate.^19,20^ However, the co-prescription of folic acid for women on valproate was found to be low (6%). These findings highlight poor adherence to clinical guidelines concerning valproate use.^4,22,32^

The standard guidelines also recommend that WRA should be on a long-acting reversible contraceptive and provide consent, which should be documented.^1,9,10,18^ Current contraceptive use was documented in 9% of the women. Furthermore, none of the records documented risk acknowledgement forms for valproate use. This is similar to other studies; one study documented that 19% of records had documented consent for valproate use and only 12% for contraceptive use.^22^ Langan et al.^32^ reported similar insufficient levels of documented teratogenic risk (22%) and family planning (13%) counselling in women on valproate. These findings strongly indicate widespread non-adherence to clinical guidelines with respect to documentation of counselling but may not be a true reflection that risk counselling was not done. Sibanyoni et al.^33^ found that more than half of the interviewed women were aware of the risk associated with valproate use and most of them reported being informed by their treating doctors.

Similarly, Galvin and Nel^6^ reported a higher prevalence (26.8%) of family planning education provided by clinicians. This shows that clinicians have discussions with patients but do not document them.

### Strengths and limitations

This retrospective study design had several strengths. It was cost-effective and time efficient. Reviewing existing clinical records provided insights into the routine clinical practice. The results obtained may form a foundation for future research and contribute towards improving clinical practice. However, the study was largely dependent on the accuracy and completeness of documented information in the files, and inconsistent documentation may have affected the validity of the findings. As the current study was conducted at an academic institution, where clinicians undergo rigorous training and continuous education, it was assumed that the clinicians at HJH are well versed in the DSM-5-TR and have accurately applied it while making the diagnoses and recording notes in the file.

The current study focused on the general prescribing approach in WRA, a specific subgroup of patients. However, it did not consider the BD subtype, the mood episode being treated or the factors influencing medication choices. Medication data were obtained from outpatient prescription charts; therefore, it was assumed that most patients were in the maintenance phase of the illness. In addition, only the most recent clinical notes and prescription charts were reviewed for documentation of counselling, contraception use, and pregnancy status. Consequently, information recorded during previous consultations was not analysed, which may have introduced potential bias into the study findings. Furthermore, inferential statistical analysis was not meaningful because of the highly skewed comparison groups, thereby limiting the ability to conduct robust statistical comparisons. Lastly, as a single-site, cross-sectional study, the results cannot be generalised to other institutions.

### Recommendations

It is recommended that clinicians consistently document reproductive health discussions such as pregnancy status, family planning and teratogenic risk associated with specific psychotropic medications. Informed consent should also be documented, and treatment guidelines closely adhered to when valproate is prescribed.

## Conclusion

This study revealed that the use of multiple psychotropic medications is common in WRA with BD. Also, a combination of valproate and an atypical antipsychotic is frequently prescribed. However, adherence to valproate-specific recommendations was suboptimal, with low documentation of contraception use, folic acid co-prescription and prior informed consent at the most recent consultation. There were also low levels of documented pregnancy status observed. These findings highlight the need for careful consideration of treatment choices to reduce the risk of potential teratogenic medication exposure.
